# Utility of nuclear stress imaging for detecting coronary artery bypass graft disease

**DOI:** 10.1186/1471-2261-12-62

**Published:** 2012-08-03

**Authors:** Basel Al Aloul, Mackenzi Mbai, Selcuk Adabag, Santiago Garcia, Hoang Thai, Steven Goldman, William Holman, Gulshan Sethi, Rosemary Kelly, Herbert B Ward, Edward O McFalls

**Affiliations:** 1 University of Minnesota, Minneapolis, MN, USA; 2 Veterans Affairs’ Medical Center, Minneapolis, MN, USA; 3 Veterans Affairs’ Medical Center, Tucson, AZ, USA; 4 Veterans Affairs’ Medical Center, Birmingham, AL, USA

**Keywords:** Coronary artery bypass grafts, CABG, Coronary artery imaging, Cardiac catheterization/intervention

## Abstract

**Background:**

The value of Single Photon Emission Computed Tomography stress myocardial perfusion imaging (SPECT-MPI) for detecting graft disease after coronary artery bypass surgery (CABG) has not been studied prospectively in an unselected cohort.

**Methods:**

Radial Artery Versus Saphenous Vein Graft Study is a Veterans Affairs Cooperative Study to determine graft patency rates after CABG surgery. Seventy-nine participants agreed to SPECT-MPI within 24 hours of their coronary angiogram, one-year after CABG. The choice of the stress protocol was made at the discretion of the nuclear radiologist and was either a symptom-limited exercise test (n = 68) or an adenosine infusion (n = 11). The SPECT-MPI results were interpreted independent of the angiographic results and estimates of sensitivity, specificity and accuracy were based on the prediction of a graft stenosis of ≥70% on coronary angiogram.

**Results:**

A significant stenosis was present in 38 (48%) of 79 patients and 56 (22%) of 251 grafts. In those stress tests with an optimal exercise heart rate response (>80% maximum predicted heart rate) (n = 26) sensitivity, specificity and accuracy of SPECT-MPI for predicting the graft stenosis was 77%, 69% and 73% respectively. With adenosine (n = 11) it was 75%, 57% and 64%, respectively. Among participants with a suboptimal exercise heart rate response, the sensitivity of SPECT-MPI for predicting a graft stenosis was <50%. The accuracy of SPECT-MPI for detecting graft disease did not vary significantly with ischemic territory.

**Conclusions:**

Under optimal stress conditions, SPECT-MPI has a good sensitivity and accuracy for detecting graft disease in an unselected patient population 1 year post-CABG.

## Background

Coronary artery bypass graft (CABG) surgery reduces angina and prolongs life in patients with severe coronary heart disease, however, the long-term effectiveness of this procedure is limited by thrombosis and accelerated atherosclerosis of the bypass grafts. It has been reported that approximately 15% of saphenous venous grafts occlude in the first year after CABG surgery and that the majority of these occlusions are clinically silent [1]. By the 6^th^ and 10^th^ year after CABG, vein graft patency rates fall to approximately 75% and 60%, respectively, [1,2] accompanied by recurrence of angina and clinical events [3]. However, symptom status is not a reliable indicator of significant graft disease with a reported sensitivity and specificity of 60% and 20%, respectively [4]. Thus, a non-invasive test to detect asymptomatic coronary artery bypass graft disease could be clinically useful.

Single photon emission computed tomography myocardial perfusion imaging (SPECT-MPI) has been shown to be an accurate test for detecting threatened bypass graft disease in patients presenting with unstable symptoms [5,6] and provides important prognostic information after CABG surgery [5-9]. However, despite its widespread acceptance in clinical practice, whether the celebratory results of SPECT-MPI in unstable patients can be extrapolated to all patients with prior CABG regardless of their symptom status is unclear.

Accordingly, the aim of this study was to assess the sensitivity, specificity and accuracy of stress SPECT-MPI in detecting coronary bypass graft disease in an unselected group of patients who underwent routine coronary angiogram one year after CABG per the protocol of the Veterans Affairs’ (VA) Cooperative trial on patency of radial artery and saphenous vein grafts [10].

## Methods

The Human Studies Subcommittee of the Research and Development committee at the Minneapolis VA Medical Center and the Executive Committee of the VA Cooperative Study (CSP #474) approved this study. All patients gave informed consent. The design was a prospective analysis of a cohort enrolled from the Minneapolis VA Medical Center who participated in the multicenter VA Cooperative trial on radial artery versus saphenous vein graft patency rates trial (NCT0005847) [10]. All subjects within the sub-study agreed to undergo either exercise or pharmacologic stress SPECT-MPI within 24 hours of their follow-up coronary angiography, which was scheduled at one year following CABG.

### Nuclear stress test

Among those patients who were deemed suitable for a treadmill exercise test by the Nuclear Radiologist, a standard Bruce protocol was performed. Patients were allowed to exercise until an end-point was reached (moderate to severe angina, shortness of breath, fatigue or leg weakness, ≥2 mm ST-segment depression, hypotension, or severe arrhythmias). Technetium-99 m tetrofosmin (Myoview) was injected at near-peak exercise, and the patients were asked to continue exercise for an additional minute. Electrocardiogram (ECG) tracings were recorded at 30-second intervals and continued into recovery, until the heart rate returned to baseline. Heart rate and blood pressure measurements were performed at rest, with each change in stage, and at peak exercise. In those patients who were not deemed candidates for exercise based on review of their clinical history, a pharmacologic stress test was performed with adenosine. In advance, they were asked to avoid consumption of any products containing methylxanthines, including coffee, tea or other caffeinated beverages, caffeine-containing drug products, and theophylline for at least 12 hours prior to the testing. A 12-lead ECG tracing and a blood pressure measure were recorded every minute during the infusion until 5 minutes following recovery. The Adenosine infusion was given at a rate of 140 mcg/kg/min over 3 minutes.

All images were acquired and processed according to the American Society of Nuclear Cardiology guidelines using one-day rest/stress protocol. The radiotracer technetium-99 m tetrofosmin (Myoview) was administered based on body weight. For the rest portion of the study, a low dose (one-fourth of the total dose, or 8–12 mCi) was given and for the stress portion, a larger dose (three-fourths of the total dose, or 24–36 mCi) was administered. Among the patients who underwent an exercise stress test protocol, the rest dose of technetium-99 m tetrofosmin was given intravenously followed by a 30 minute waiting period, after which the first set of images was acquired over 15 minutes. After a short delay, the exercise stress test was performed and the dose of technetium-99 m tetrofosmin was given 1 minute before the completion of exercise. After a 45-minute waiting period, a second set of images was acquired and collected over 20 minutes. Among the patients who underwent the Adenosine stress protocol, the rest dose of technetium-99 m tetrofosmin was given intravenously followed by a 30 minutes waiting period after which the first set of imaging was performed. After a short delay, the six-minute Adenosine infusion was begun and at 3 minutes, the stress dose of technetium-99 m tetrofosmin was injected. After a 45-minute delay, a second set of images was acquired.

All raw data of gated SPECT images were reconstructed using standard back-projection and identical filtering. Quantitative SPECT was performed using a previously validated automated program that determines the extent and severity of left ventricle perfusion defect size and the extent of reversible (ischemia) or fixed (scar) perfusion defects [11]. In addition, the automated program was used to derive the summed stress score (SSS), summed rest score (SRS), and summed difference score (SDS) based on conventional 17-segment model. The program assigned a score of 0 to 4 to each segment based on activity level: 0 = normal and 4 = absent. In addition to perfusion data, the left ventricular ejection fraction, end-diastolic volume, and end-systolic volume were measured from the gated SPECT as previously described [11]. The SPECT images were interpreted by 2 experienced readers according to a standard segment scoring system that provides semi-quantitative information regarding total number of defects and the degree of ischemia versus infarction. The agreement between the 2 readers was 82% with a Kappa value of 0.62 [12]. The interpretation of the SPECT was made without knowledge of the results of the coronary angiogram.

### Coronary angiography

Coronary angiography was performed one-year post CABG and within 24 hours of the SPECT-MPI. All cine-angiograms were performed using standard techniques, including nitroglycerin administration, in at least 2 orthogonal angiographic views (45°left anterior oblique and 45° right anterior oblique). All angiograms were interpreted at the core laboratory, blinded to the results of the SPECT-MPI, using quantitative coronary angiography per the VA Cooperative study protocol [10]. A cutoff of stenosis severity ≥70% was used to define graft disease.

### Statistical analysis

Continuous variables were displayed as mean ± one standard deviation and categorical variables as percentages. Prevalence of chronic obstructive pulmonary disease (COPD) in patients with versus without adequate heart rate response to exercise was compared with chi-square test. Statistical analyses were performed using SPSS version 16. A two-tailed p value of < 0.05 was considered statistically significant. Invasive bypass angiography served as the “gold standard” to define the presence of graft disease. The sensitivity, specificity, positive predictive value, negative predictive value and accuracy of SPECT'-MPI to detect significant graft disease on coronary angiography were calculated as follows [13]. Sensitivity is defined as the number of true positives divided by the combined number of true positives and false negatives. Specificity is the number of true negatives divided by the combined number of true negatives and false positives. Positive predictive value is the number of true positives divided by the combined number of true positives and false positives. Negative predictive value is the number of true negatives divided by the combined number of true negatives and false negatives.

Cohen’s kappa coefficient (κ) is a statistical measure of inter-rater agreement. It is calculated as the relative observed agreement among readers minus the hypothetical probability of chance agreement divided by 1 minus the hypothetical probability of chance agreement. If the raters are in complete agreement then κ = 1. If there is no agreement among the raters other than what would be expected by chance, then κ = 0 [12].

## Results

From February 2003 through February 2008, 82 patients were enrolled in the VA Cooperative sub-study and 79 patients were included in the final analysis. Three patients were excluded because one declined coronary angiography, one had exercise stress test in the presence of left bundle branch block and one had Thallium tracer instead of Technetium tracer during SPECT-MPI. The majority of study participants were male with an average age of 60 ± 7 years (Table 1).

**Table 1:** Baseline characteristics of study participants

	
Age (years) ± SD	60 ± 7
Male Gender (%)	78 (99)
BMI (kg/m^2^) ± SD	30 ± 6
Arterial hypertension (%)	69 (87)
Chronic obstructive pulmonary disease (%)	19 (24)
Cerebrovascular disease (%)	9 (11)
Diabetes mellitus (%)	23 (29)
Current smoking (%)	22 (28)
*Surgery – CABG*	
Priority	
Elective (%)	74 (94)
Urgent (%)	5 (6)
*Grafts*	
LIMA (%)	77 (97)
Radial Artery	37 (47)
≥ 1 Saphenous Vein Graft (No Radial Artery Graft)	42 (53)
*Total Number of Grafts*	
Two (%)	15 (19)
Three (%)	36 (46)
Four (%)	28 (35)

### Coronary angiography

At the scheduled coronary angiogram at 1-year post-CABG, 38 of the 79 (48%) patients had significant graft disease (≥70% stenosis, including total occlusion) involving 56 of 251 (22%) potential grafts. There was 77 left internal mamary grafts and of those 6 had significant disease (7.8%). A total of 37 radial artery grafts were used and of those 12 had significant disease (32%). Lastly, 137 saphenous vein grafts were used and of those 38 grafts had significant disease (28%). A complete graft occlusion was present in 33 (13%) of 251 grafts.

### Nuclear stress test

Exercise SPECT-MPI was the designated stress protocol in 68 (86%) of the 79 study patients. As a group, the heart rate at peak exercise was 122 ± 18 beats per minute and the total exercise time was 8 ± 3 minutes. The peak double product was 19,912 ± 4,310 and the mean maximum predicted heart rate (MPHR%) was 80% ± 20%. Among those patients who underwent symptom-limited exercise, the test was adequate (i.e. MPHR > 80%) in 26 patients (38%) and inadequate (i.e. MPHR ≤ 80%) in 42 patients (62%). Pharmacological SPECT-MPI was the designated stress protocol in 11 (14%) of the 79 patients. Among patients with a suboptimal exercise performance, a higher prevalence of chronic obstructive pulmonary disease was noted compared with individuals with an adequate exercise performance (82% versus 18%; p < 0.05).

### Detection of graft disease with SPECT

**Figure 1:**
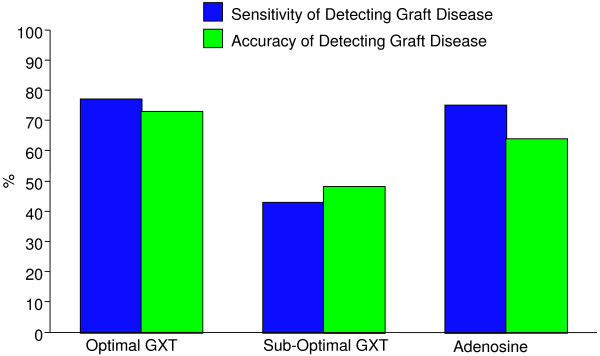
The sensitivity of detecting graft disease at 1-year following CABG is shown according to the stress protocol and achievement of adequate heart rate response.

An abnormal SPECT-MPI was present in 42 (53%) of the 79 patients, including 14 patients with an adequate exercise heart rate response, 21 patients with an inadequate exercise heart rate response and 7 patients with a pharmacological stress protocol (Table 2). An adequate exercise stress SPECT-MPI protocol, with an ability to achieve >80% of the MPHR had a sensitivity of 77%, specificity of 69%, positive predictive value of 71% and negative predictive value of 75% for the detection of graft disease (Table 3). On the other hand, an inadequate exercise stress SPECT-MPI protocol, with an inability to achieve >80% of the MPHR had a sensitivity for detecting a graft stenosis of 43%, with a specificity of 52%, positive predictive value of 47% and negative predictive value of 48%. Conversely, pharmacological stress SPECT-MPI had a sensitivity for detecting graft disease of 75%, with a specificity of 57%, positive predictive value of 50% and negative predictive value of 80% (Figure 1).

**Table 2:** Results of nuclear stress test

***Nuclear Stress Test***	
Exercise (Tc-99 m) -number of patients (%)	68 (86)
Adenosine (Tc-99 m) –number of patients (%)	11 (14)
Peak Heart Rate (bpm) ± SD	122 ± 18
Exercise Time (minutes) ± SD	8 ± 3
Peak Double Product ± SD	19,912 ± 4310
SRS ± SD	5 ± 8
SSS ± SD	6 ± 8
SDS ± SD	1 ± 2
*Type of Perfusion Defect*	
No Defects (%)	37 (47)
Reversible (%)	7 (9)
Fixed (%)	23 (29)
Mixed (%)	12 (15)
Ejection Fraction ± SD	53 ± 12
End Systolic Volume (cc^3^) ± SD	59 ± 41
End Diastolic Volume (cc^3^) ± SD	115 ± 53
Total Dose (mCi) ± SD	47 ± 7

*Bpm*: beats per minute. *TC*: Technetium. *SRS*: Sum rest score. *SSS*: Sum stress score. *SRS*: Sum rest score. *SDS*: Sum difference score. *mCi*: Millicurie.

**Table 3:** Performance of single photon emission computed tomography myocardial perfusion imaging (SPECT-MPI) stress testing for detecting coronary artery bypass graft patency

**Stress test**	**Stress test adequacy**	**Sensitivity (%)**	**Specificity (%)**	**PPV (%)**	**NPV (%)**
**Exercise SPECT-MPI (n = 68)**	**Adequate: MPHR > 80% (n = 26)**	77	69	71	75
	**Inadequate: MPHR ≤ 80% (n = 42)**	43	52	47	48
**Pharmacologic SPECT-MPI (n = 11)**		75	57	50	80

*MPHR*: Maximum predicted heart rate. *PPV*: Positive predictive value. *NPV*: Negative predictive value.

The accuracy of SPECT-MPI varied slightly by the myocardial territory supplied by the bypass graft (Table 4). An adequate exercise stress SPECT-MPI protocol had an accuracy of 81%, 76% and 78% for grafts supplying the anterior, lateral and inferior walls, respectively. On the other hand, adenosine stress protocol had an accuracy of 55%, 80% and 63% for grafts supplying the anterior, lateral and inferior walls, respectively.

**Table 4:** Accuracy of single photon emission computed tomography myocardial perfusion imaging (SPECT-MPI) stress testing for detecting coronary artery bypass graft disease according to the territory that is supplied by the diseased graft

**Stress test**	**Stress test adequacy**	**Accuracy-Overall (%)**	**Accuracy-Anterior (%)**	**Accuracy-Lateral (%)**	**Accuracy-Inferior (%)**
**Exercise SPECT-MPI (n = 68)**	**Adequate: MPHR > 80% (n = 26)**	73	81	76	78
	**Inadequate: MPHR ≤ 80% (n = 42)**	48	83	55	54
**Pharmacologic SPECT-MPI (n = 11)**		64	55	80	63

## Discussion

In this prospective cohort study of unselected patients returning for a coronary angiogram at one-year following CABG, SPECT-MPI was shown to have a good sensitivity, specificity, and accuracy for detecting bypass graft disease with an adequate exercise or pharmacological stress test. However, in those individuals who failed to achieve >80% of their maximal predictive heart rate, the sensitivity, specificity and accuracy decreased significantly. We also found that the accuracy of SPECT-MPI did not vary between different myocardial territories. To our knowledge, these are the first prospective data on the accuracy of SPECT-MPI in an unselected cohort of patients after CABG.

There are limited data on the accuracy of SPECT imaging in asymptomatic patients with prior CABG. However, in previous retrospective studies of symptomatic patients who underwent coronary angiography, the sensitivity and specificity of detecting graft disease as judged by exercise thallium-201 SPECT was 80% and 87% respectively [5]. Further, sensitivity of SPECT-MPI was significantly higher than that of the exercise electrocardiogram stress test in patients with typical recurrent angina (84% vs. 24%), as well as in those with atypical symptoms (70% vs. 50%). Moreover, among symptomatic patients who underwent coronary angiography, comparable results were shown with adenosine thallium-201 SPECT, with a sensitivity and specificity for detecting graft disease of 96% and 60%, respectively [14]. A low specificity was attributed to several factors, including perfusion abnormalities in the distribution of non-bypassed native vessels, and partial volume effects as a result of regional wall motion and conduction abnormalities [14]. Because the pretest probability of bypass graft disease in these symptomatic patients was elevated, the accuracy of the SPECT-MPI found in these studies may not necessarily be extrapolated to unselected patient cohorts. Thus the present investigation fills this gap in the literature by assessing the utility of SPECT-MPI in an unselected study cohort decreasing the chances of a selection bias.

We have found that with optimal stress conditions, SPECT-MPI has a sensitivity and a negative predictive value of >75% with either adequate exercise or pharmacological stress testing. The sensitivity is slightly lower than prior studies because of differences in symptoms at the time of presentation. At 5 years following bypass surgery, it is expected that 25% of vein grafts have significant disease and a substantial proportion of patients have symptoms, increasing the pretest probability. Further, exclusion of patients with a negative stress test in previous studies might have introduced a selection bias in the sensitivity and specificity calculations [15]. Indeed, exclusion of selected patients may curtail the number of true-negative results and consequently raises the sensitivity of a test [16].

In the present study, SPECT-MPI was more sensitive and specific for detecting bypass graft disease in patients with an adequate exercise heart rate response compared with those who did not achieve an adequate heart rate. The sensitivity of adequate exercise SPECT-MPI was 77%, in comparison to < 50% in patients with an inadequate exercise heart rate response. These data underscore the importance of continuing exercise stress testing until MPHR is achieved or opting for adenosine protocol in patients who are unlikely to achieve adequate exercise.

Until now invasive bypass graft angiography remains the gold standard for detecting graft disease [17,18]. As shown in this study SPECT-MPI is useful to detect graft disease accurately. Recently, it has been shown that combining perfusion CT imaging and cardiac CT angiography is feasible, and CT perfusion adds incremental value to cardiac CT angiography in the detection of significant coronary artery disease [19,20], but these studies were not performed specifically in patients with bypass grafts.

In our study 38 of the 79 (48%) patients had significant graft disease (≥70% stenosis, including total occlusion) involving 56 of 251 (22%) potential grafts, which is slightly higher than previously reported. It is known that approximately 15% of saphenous venous grafts occlude in the first year after CABG surgery and that the majority of these occlusions are clinically silent [1]. Potential explanations for these differences are considered. First, prior studies have reported more significant stenosis or complete occlusion to define graft disease, whereas we reported any stenosis > 70%. Of note a complete graft occlusion was present in 33 (13%) of 251 grafts, which is similar to previous reports. Second in a study with a small sample size like ours, a few patients can alter the proportion with significant lesions. Third, the fact that 99% of patients were male and 28% of them were active smokers might have contributed to the slight increase in graft disease.

The most notable strength of this investigation is its prospective study design. Indeed, all of the previous information in this area has originated from retrospective analyses in which potential bias has been created in referral to either the nuclear stress lab and/or coronary angiography. In addition to the prospective nature and the all inclusiveness of the participants, an additional strength of the present study is that the cardiac catheterization and the nuclear imaging tests were performed within 24 hours of each other and both tests were interpreted without knowledge of the results of the other. In previous studies, these examinations were interpreted weeks and sometimes months apart, during which changes in coronary anatomy or perfusion might have occurred. This study also has some limitations. First, 42 out of 68 patients who performed an exercise stress SPECT did not achieve the MPHR. Chronic beta-blocker use and high prevalence of non-cardiac co-morbidities such as COPD are the most likely explanation for this finding. This observation raises the issue of whether a pharmacological stress should be considered the preferred modality in this patient population. Second, it might be difficult to assess the perfusion defects based on a cut of stenosis severity focally due to distal circulation status and collateral vessels. It is possible that some perfusion defects were due to preexisting distal vessel disease. Third, the sample size is small in statistical terms. Fourth, nearly all patients were male with high prevalence of COPD. Caution should be exercised in extrapolating these results to women and the study findings require confirmation in other populations with a lower prevalence of COPD.

## Conclusions

In this prospective cohort study of an unselected cohort of patients at one year following CABG, SPECT-MPI showed a good sensitivity, specificity, and accuracy for detecting angiographic bypass graft disease under optimal stress conditions.

## Abbreviations

SPECT-MPI: Single Photon Emission Computed Tomography stress myocardial perfusion imaging; CABG: Coronary Artery Bypass Graft; COPD: Chronic Obstructive Pulmonary Disease; VA: Veterans Affairs’; ECG: Electrocardiogram; SSS: Summed Stress Score; SRS: Summed Rest Score; SDS: Summed Difference Score; MPHR: Maximum Predicted Heart Rate.

## Competing interests

All authors declare that they have no competing interests.

## Authors’ contributions

All authors participated in the study design, coordination, and data acquisition and analysis, and helped to draft the manuscript. All authors read and approved the final manuscript.

## Pre-publication history

The pre-publication history for this paper can be accessed here:

http://www.biomedcentral.com/1471-2261/12/62/prepub
